# Associations between IL-1α, IL-1β, TNFα, and IL-6 variations, and susceptibility to transposition of the great arteries

**DOI:** 10.1186/s12872-022-02670-1

**Published:** 2022-05-19

**Authors:** Latife Atasoy Karakas, Duygu Tugrul, Nihal Sahin Uysal, Sertac Esin, Niyazi Kursat Tokel, Yunus Kasim Terzi

**Affiliations:** 1 Department of Obstetrics and Gynecology, Baskent University Faculty of Medicine, Baskent University Hospital, Sehit Temel Kugulu sok 34, 06490 Bahcelievler, Cankaya, Ankara Turkey; 2 Department of Pediatric Cardiology, Baskent University Faculty of Medicine, Ankara, Turkey; 3 Department of Medical Genetics, Baskent University Faculty of Medicine, Ankara, Turkey

**Keywords:** IL-1α -889C/T (rs1800587), IL-1β -511C > T (rs16944), TNFα -308G > A (rs1800629), TNFα -238G > A (rs361525), IL-6 -174G > C (rs1800795), IL-6 -572G > C (rs1800796), Promoter, Polymorphisms, Transposition of the great arteries

## Abstract

**Background:**

To evaluate the relationship between IL-1α -889C/T (rs1800587), IL-1β -511C > T (rs16944), TNFα -308G > A (rs1800629), TNFα -238G > A (rs361525), IL-6 -174G > C (rs1800795), and IL-6 -572G > C (rs1800796) polymorphisms and the susceptibility to transposition of the great arteries (TGA).

**Methods:**

A prospective analysis was performed on mothers whose newborns were diagnosed as having TGA. For each case of TGA, a mother who gave birth to a healthy neonate in the same period was randomly selected for the control group. The sample size was calculated before planning the study with 80% power and 5% alpha.

**Results:**

Twenty-seven mothers whose newborn had TGA anomalies (group 1) and 27 mothers whose newborn had no TGA (group 2) were included in the study. There were no significant differences between the groups in terms of maternal age, pregestational body mass index, gestational age at birth and infant sex (*p* > 0.05). The genotype and allele distributions of IL-1α -889C/T (rs1800587), IL-1β -511C > T (rs16944), TNFα -308G > A (rs1800629), TNFα -238G > A (rs361525), IL-6 -174G > C (rs1800795) and IL-6 -572G > C (rs1800796) gene variants were not different between the two groups (*p* > 0.05).

**Conclusions:**

There was no relation between IL-1α, IL-1β, IL-6, and TNFα promoter gene polymorphisms and TGA occurrence in our study group.

*Trial registration*: This present prospective case–control study was conducted in Baskent University Hospital, Ankara, Turkey, between May 2020 and November 2021. Ethical approval was obtained from the university’s Clinical Research Ethics Commitee (No: KA20/211) in accordance with the Declaration of Helsinki.

## Background

The prevalence of congenital heart disease (CHD) is 8–10 in 1000 live births [1]. CHD is the most common structural anomaly of the newborn and is defined as anatomic malformations of the heart or great vessels [1, 2]. Cardiac anomalies are classified as cyanotic and acyanotic heart diseases, depending on the outflow of vessels from the heart and structural dysfunction in the heart [1, 2]. Transposition of the great arteries (TGA) is the most common cardiac anomaly among cyanotic heart diseases [3]. The etiologic origin of these anomalies is unknown in 85% of cases; however, genetic abnormalities, mesenchymal tissue migration errors, intracardiac blood flow defects, extracellular matrix abnormalities, and apoptosis may explain the developmental mechanism [4–6].

During the first weeks of pregnancy, mesenchymal stem cells migrate and differentiate into vascular and cardiac smooth muscle cells [7, 8]. Proinflammatory cytokines such as interleukin-1 (IL-1), interleukin-6 (IL-6), and tumor necrosis factor-alpha (TNFα) promote this migration and differentiation [9, 10]. If this process is interrupted, incomplete embryonic development can result in CHD. Polymorphisms in the promoter region of a gene increase transcription of that gene by altering the functioning of the promoter [11]. Growing evidence suggests that the promoter polymorphism of the proinflammatory cytokines is closely associated with CHD susceptibility [11–13]. Moreover, the expression of the polymorphism in different types of CHD is probably different [14]. To our knowledge, the role of cytokine polymorphisms has not been studied in TGA development. Frequently studied cytokine polymorphisms which have been shown to be related to cardiovasculer diseases and located promotor region of gene are IL-1α -889C/T (rs1800587), IL-1β -511C > T (rs16944), TNFα -308G > A (rs1800629), TNFα -238G > A (rs361525), IL-6 -174G > C (rs1800795), and IL-6 -572G > C (rs1800796) [11, 12, 15, 16]. In this study, these promotor polymorphisms have been chosen to explore their association with TGA risk.

The aim of this study was to evaluate the relationship between IL-1α -889C/T (rs1800587), IL-1β -511C > T (rs16944), TNFα -308G > A (rs1800629), TNFα -238G > A (rs361525), IL-6 -174G > C (rs1800795), and IL-6 -572G > C (rs1800796) promoter region polymorphisms and susceptibility to TGA in our sample group.

## Methods

This present prospective case–control study was conducted in Baskent University Hospital, Ankara, Turkey, between May 2020 and November 2021. Ethical approval was obtained from the university’s Clinical Research Ethics Commitee (No: KA20/211).

The newborns whose cardiac anomalies were detected during their antenatal follow-up and diagnosed as TGA after birth with an echocardiography performed by a pediatric cardiology specialist were included in the study. Neonates with TGA associated with chromosomal anomalies and genetic syndromes were excluded. For each cardiac anomaly case, mothers who gave birth to a healthy neonate in the same period were randomly selected as the control group. Healthy newborns with congenital disease or a family history of CHD or other heart diseases were excluded. Women with a history of immigration or marriage from other nationalities within three generations, who had a history of diabetes mellitus, phenylketonuria, exposure to radiation, teratogen or chemicals during pregnancy were excluded.

Written consent from each participant was obtained. Volunteers were invited to collect 5 mL of venous blood samples from the umbilical cord into an anticoagulative tube with EDTA-disodium salt at birth.

Genomic DNA was extracted from all patients and control individuals. IL-1α -889C/T (rs1800587), IL-1β -511C > T (rs16944), TNFα -308G > A (rs1800629), TNFα -238G > A (rs361525), IL-6 -174G > C (rs1800795), and IL-6 -572G > C (rs1800796) promoter region polymorphisms were analyzed using polymerase chain reaction (PCR) and the SNaPhot method. Primer sequences for PCR and SNaPhot analysis are given in Table 1. PCR conditions were 5 min at 95 °C for initial denaturations, followed by 35 cycles of 1 min at 94 °C, 1 min at 58 °C for IL-1α and TNFα, and at 56 °C for IL-1β and IL-6, and 1 min at 72 °C. The PCR was completed after a 10 min final elongation step at 72 °C. The final concentrations of the PCR contents were 0.4 μM of each primer, 0.1 mM of dNTP (Thermofisher, MA USA), and 0.03 U/μL of Hot Start Taq DNA polymerase (NEB).

**Table 1:** Primer sequences for PCR and amplicon lengths and SNaPhot primers were used in this study

SNP	Forward Primer (5′–3′)	Reverse Primer(5′–3′)	Size (bp)	Tm (°C)
*PCR primers*				
IL-1α	GCTTCACTATGTTGCCCACA	CAGTAAAGTAGCCCTCTACCAA	462	58
IL-1β	GGCATTGATCTGGTTCATCC	GTTTAGGAATCTTCCCACTT	304	56
TNFα	GAAACAGACCACAGACCTGG	ACACAAGCATCAAGGATACC	177	58
IL-6	CACGAAATTTGAGGATGGCC	GGCTGATTGGAAACCTTATT	489	56
*SNaPshot primers*				
IL-1α, rs1800587	ATAATAGTAACCAGGCAACA	TTACATATGAGCCTTCAATG		
IL-1β, rs16944	TGCAATTGACAGAGAGCTCC	TGGGTGCTGTTCTCTGCCTC		
TNFα, rs1800629	AATAGGTTTTGAGGGGCATG	CTGGAGGCTGAACCCCGTCC		
TNFα, rs361525	AGAAGACCCCCCTCGGAATC	ACTCCCCATCCTCCCTGCTC		
IL-6, rs1800795	TCCCCCTAGTTGTGTCTTGC	AATGTGACGTCCTTTAGCAT		
IL-6, rs1800796	CAGGCAGTTCTACAACAGCC	GTTCTGGCTCTCCCTGTGAG		

*bp* base pair

The SNaPshot method was used for genotyping purposes. PCR products were purified by using ExoSAP according to the manufacturer protocols (Affymetrix Inc., Santa Clara, CA, USA). Briefly, 5μL PCR products were mixed with 2μL ExoSAP. The mixture was incubated at 15 min at 37 °C and 15 min at 80 °C. Purified PCR products were used in SNaPshot reactions according to the manufacturer’s protocols (SNaPshot Multiplex Kit, ABI Prism, Foster City, CA, USA). SNaPshot conditions were 1 min at 96 °C for initial denaturations followed by 25 cycles of 10 s at 96 °C, 5 s at 50 °C, and 30 s at 60 °C. Final concentrations of the SNaPshot contents were 0.2 μM of each primer, 1X of SNaPshot Multiplex Ready Reaction Mix, and 3μL of purified PCR products. SNaPshot products were run on the ABI Prism 3130 Genetic Analyzer (ABI Prism, Foster City, CA, USA). The results were analyzed using the Genemapper Software v4.0 (ABI Prism, Foster City, CA, USA).

The cases included in the study were divided into two groups: mothers of infants with TGA anomalies (group 1) and mothers of infants without TGA (group 2).

The sample size was calculated when planning the study. If an effect size of 0.80 was desired, according to Student’s t-test, it was found that at least 52 participants (at least 26 participants in each group) must be included in the study to test the statistical significance of the differences between the groups (with TGA and without TGA) with 80% power and 5% alpha.

Data were analyzed using the SPSS 24.0 software package (IBM Corp., Armonk, NY, USA). The variables were investigated using the Kolmogorov–Smirnov or Shapiro–Wilk test to determine whether they were normally distributed. The inferential statistics tests used were the independent Chi-square (χ^2^) test and Fisher exact test for categorical data. For non-normally-distributed variables, descriptive analyses are presented using median values. The Mann–Whitney *U* test was conducted to compare these parameters. An overall 5% type-I error level was used to infer statistical significance.

## Results

Fifty-four volunteers who met the study criteria were in included the study. Of these participants, 27 were mothers of newborns with TGA anomalies (group 1), and 27 mothers of newborns with no TGA anomalies (group 2). The mean maternal age of the study population was 31 (range, 25–39) years. The demographic data and descriptive characteristics of the groups are presented in Table 2. There were no significant differences between the groups in terms of maternal age, pregestational body mass index (BMI), gestational age at birth and infant sex (*p* = 0.335, *p* = 0.320, *p* = 0.161, and *p* = 0.412, respectively).

**Table 2:** Demographic data and descriptive characteristics of the groups

Characteristics	With TGA n = 27 group 1	Without TGA n = 27 group 2	*p*-value
Maternal age, year	32 (25–39)	31 (25–36)	0.335*
Pregestational BMI (kg/m^2^)	21.4 (18–37)	21.1 (19–32)	0.320*
Gestational week at birth	38 (36–39)	39 (36–40)	0.161*
Infant sex			0.412**
Girl	10 (38.5)	14 (51.9)	
Boy	16 (61.5)	13 (48.1)	

^†^Values are presented as median (minimum–maximum) or number (%)

*Mann–Whitney U test

**X^2^ test

IL-1α -889C/T (rs1800587), IL-1β -511C > T (rs16944), TNFα -308G > A (rs1800629), TNFα -238G > A (rs361525), IL-6 -174G > C (rs1800795), and IL-6 -572G > C (rs1800796) genotype and allele frequencies of genes were compared between groups. There was no significant difference in genotypes and allele distributions of each promoter region between the two groups (*p* = 0.713, *p* = 0.831, *p* = 0.553, *p* = 0.999, *p* = 0.999, and *p* = 0.999, respectively). Table 3 summarizes the comparison of proinflammatory cytokine polymorphisms between the groups.

**Table 3:** Comparison of pro-inflammatory cytokine polymorphism between groups

Characteristics	With TGA n = 27 group 1	Without TGA n = 27 group 2	*p*-value
IL-1α -889C/T (rs1800587)			0.713*
CC	15 (55.6)	12 (44.4)	
CT	8 (29.6)	11 (40.7)	
TT	4 (14.8)	4 (14.8)	
C	38 (70.4)	35 (64.9)	
T	16 (29.6)	19 (35.1)	
IL-1β -511C > T (rs16944)			0.831*
CC	10 (37)	8 (29.6)	
CT	13 (48.1)	14 (51.9)	
TT	4 (14.8)	5 (18.5)	
C	33 (61.2)	30 (55,6)	
T	21(38.8)	24 (44.4)	
TNFα -308G > A (rs1800629)			0.553*
AA	0 (0)	1 (3.7)	
GA	5 (18.5)	6 (22.2)	
GG	22 (81.5)	20 (74.1)	
A	5 (9.3)	8 (14.9)	
G	49 (90.7)	46 (85.1)	
TNFα -238G > A (rs361525)			0.999*
AA	0 (0)	0 (0)	
GA	3 (11.1)	3 (11.1)	
GG	24 (88.9)	24 (88.9)	
A	3 (5,6)	3 (5,6)	
G	51 (94.4)	51 (94.4)	
IL-6 -174G > C (rs1800795)			0.999*
GC	8 (29.6)	9 (33.3)	
GG	19 (70.4)	18 (66.7)	
CC	0 (0)	0 (0)	
G	46 (85.1)	45 (83.3)	
C	8 (14.9)	9 (16.7)	
IL-6 -572G > C (rs1800796)			0.999*
GC	6 (22.2)	5 (18.5)	
GG	21 (77.8)	22 (81.5)	
CC	0 (0)	0 (0)	
G	48 (88.8)	49 (90.7)	
C	6 (11.2)	5 (9.3)	

Values are presented as number (%)

*X^2^ test

## Dıscussıon

It is believed that this is the first study in the literature to analyze the relation between the polymorphisms in the promoter regions of IL-1α -889C/T (rs1800587), IL-1β -511C > T (rs16944), TNFα -308G > A (rs1800629), TNFα -238G > A (rs361525), IL-6 -174G > C (rs1800795), and IL-6 -572G > C (rs1800796) genes, and the susceptibility to TGA congenital cardiac anomalies. The results demonstrated that these single-nucleotide polymorphisms had no relation with TGA susceptibility.

TNFα is a pleiotropic pro-inflammatory cytokine, which is a mediator of many immunologic and metabolic effects and stimulates the production of other proinflammatory cytokines such as IL-6 and IL-1 [17]. Many studies have focused on two variants of promoter gene polymorphisms of TNFα such as -308G > A (rs1800629) and TNFα -238G > A, that were found to affect TNFα production in CHD and chronic diseases [12, 18]. Although they could not fully investigate the effect of these polymorphism on transcription factors, Jun Pan et al. studied the association of the TNFα -308G > A (rs1800629) gene polymorphism with susceptibility to CHD [12]. They suggested that the G allele was observed more frequently (74.2%) than the A allele (25.7%) in congenital hearth disease. They found that the A allele as a risk factor for CHD and can be used as a potential non-invasive biomarker to detect congenital hearth disease in the Chinese population. Similar to their study, the most common allele in our TGA population was the G allele (90.7%). However, TNFα -308G > A (rs1800629) variant genotypes were similar between groups, and A allele frequency was not related to TGA susceptibility in our population. We could not reach the number of TGA cases in the congenital cardiac anomaly population in their studies, but it should be noted that our population consisted of only patients with TGA anomalies. Additionally, the small size of the patient population with the A allele genotype in our study may be responsible for this difference.

The IL-6 polymorphism has also been associated with heart and chronic diseases [11, 19]. Zang et al. checked the genotype frequencies for the variants IL-6 -174G > C (rs1800795) and IL-6 -572G > C (rs1800796) in CHD and control groups [11]. They found a close association between IL-6 -174G > C (rs1800795) carriage and CHD susceptibility in the Chinese population. They also found that the incidence of the mutant C allele was high in the CHD group. In their study, the IL-6 -174G > C (rs1800795) variant had a 28.4% C allele frequency in the CHD group, whereas this rate was 14.9% in our study. We found no association between the IL-6 -174G > C (rs1800795) variant and TGA susceptibility. This difference may be due to the low frequency of C alleles in our study. The effect of the rs1800795 promoter gene polymorphism on CHD onset risk is still not clear. The distribution of HLA alleles on cytokine polymorphisms may differ in different geographic areas and different populations.

The IL-1 gene family, which includes the pro-inflammatory cytokines IL-1α and IL-1β, is a key mediator for the regulation of apoptosis [20]. Genetic variations in the IL-1 gene family affect pro-inflammatory gene regulation and have been associated with high levels of proinflammatory mediators [16, 21, 22]. To date, IL-1α and IL-1β promoter regions of gene polymorphisms have been associated with a variety of chronic and heart diseases [15, 16, 23]. However, these gene polymorphisms have not yet been associated with congenital cardiac anomalies. In a study from Turkey, the IL-1β -511C > T (rs16944) polymorphism was determined to be associated with nasal polyposis [24]. The frequency of the T allele in that study was 38% and 46% in the case and control groups, respectively. However, the T allele frequency was 29.6% in our sample group. The reason why we could not establish a significant relation between IL-1β -511C > T (rs16944) polymorphism and TGA susceptibility in our study may be due to the lower frequency of T alleles compared with their study’s sample group. It may also be due to other genetic causes, such as expression steps of gene transcription or single-gene mutations.

This study has some limitations. First, we could not reach the effect of these polymorphisms on cytokine levels because the protein level of cytokines in the peripheral blood was not detected. Second, other unknown etiologic factors such as environmental factors or other genetic factors in TGA have not been investigated. Third, although this study was planned using power analysis, the sample size we evaluated may not reflect the allele frequency and genotype distributions in the entire population.

In conclusion, TGA is a serious disease that has a financial and moral burden for both newborns’ families and society. Antenatal diagnosis is valuable because it improves the management of the unstable postpartum period including delivery room team medications, and emergency surgical planning [14, 25, 26]. Therefore, understanding the biology of the disease and translating this knowledge to the bedside is critical. We could not establish a link between IL-1, IL-6, and TNFα promoter gene polymorphisms and TGA susceptibility. However, this relation may be established at the steps of protein synthesis or gene expression. Well-designed, large-scale, randomized controlled trials are necessary to improve our understanding of the effect of cytokine polymorphism in the TGA. We think that identifying candidate genes responsible for TGA in the future will bring new perspectives to diagnosis and treatment such as gene ablation studies.

## Data Availability

The data-sets generated and/or analysed during the current study are not available because of the lack of consent from the study participants, but are available from the corresponding author on reasonable request.

## Abbreviations

TGA: Transposition of the great arteries
CHD: Congenital heart disease
IL-1: Interleukin-1
IL-6: Interleukin-6
TNFα: Tumor necrosis factor-alpha
PCR: Polymerase chain reaction
BMI: Body mass index
